# The Appendicitis Discussion of the Philadelphia County Medical Society

**Published:** 1891-12

**Authors:** 


### THE APPENDICITIS DISCUSSION OF THE PHILADELPHIA COUNTY MEDICAL SOCIETY.

WE NEED not apologize to our readers for taking up so much space in the present number of the JOURNAL with a discussion of appendicitis. In the first place, it is a subject that every physician must be familiar with, in order that he may recognize the time when the malady passes from the domain of medicine to that of surgery, and to invoke promptly experienced and competent surgical aid. It will not answer now-a-days to treat indifferently recurrent attacks of pain in the right iliac fossa, or to batten down the patient and mask the symptoms in the early stages of the disease, so that the surgeon when he arrives is placed at a disadvantage with reference to the diagnosis, or as to the necessity for operative treatment. The surgeon should early see the case in connection with the physician, not necessarily to operate at once as some have advocated, but rather to become familiar with the case that the opportune moment for operation may be seized if it should present itself, and not have the patient sacrificed because of ignorance, hesitation, or timidity.

We have published in full this remarkable discussion on the subject that took place in the Philadelphia County Medical Society. A special meeting was appointed to be held September 28th, so that Mr. Thomas Bryant, who was visiting this country, might participate therein. His views, though leaning to the so-called conservative side of the question, deserve careful consideration; but we are of the opinion that the majority of the profession, most familiar with this disease, will, in the main, approve of the position taken by Dr. Morton in this discussion, which is certainly in our present light well nigh impregnable. We bespeak a careful study of this discussion from every point of view, and particularly should the remarks of the speakers following the papers not be omitted in the reading. We could not omit any portion of it without injustice to the speakers, and to divide it with two or three issues of the JOURNAL would spoil its effect, so we have published it to the exclusion of all other original matter. Our contributors will bear with us for the slight delay it occasions in the publication of their articles, we feel sure.

